# Loss of Pten Disrupts the Thymic Epithelium and Alters Thymic Function

**DOI:** 10.1371/journal.pone.0149430

**Published:** 2016-02-25

**Authors:** Phillip M. Garfin, Thuyen Nguyen, Julien Sage

**Affiliations:** 1 Department of Pediatrics, Stanford University, Stanford, CA, United States of America; 2 Department of Genetics, Stanford University, Stanford, CA, United States of America; University of Tokyo, JAPAN

## Abstract

The thymus is the site of T cell development and selection. In addition to lymphocytes, the thymus is composed of several types of stromal cells that are exquisitely organized to create the appropriate environment and microenvironment to support the development and selection of maturing T cells. Thymic epithelial cells (TECs) are one of the more important cell types in the thymic stroma, and they play a critical role in selecting functional T cell clones and supporting their development. In this study, we used a mouse genetics approach to investigate the consequences of deleting the *Pten* tumor suppressor gene in the TEC compartment of the developing thymus. We found that PTEN deficiency in TECs results in a smaller thymus with significantly disordered architecture and histology. Accordingly, loss of PTEN function also results in decreased T cells with a shift in the distribution of T cell subtypes towards CD8^+^ T cells. These experiments demonstrate that PTEN is critically required for the development of a functional thymic epithelium in mice. This work may help better understand the effects that certain medical conditions or clinical interventions have upon the thymus and immune function.

## Introduction

As the site of T cell maturation and selection, the thymus is essential for proper function of the immune system [1]. In their migration through the thymus, T cells interact with several different cell types [2]. One of the important cell types, thymic epithelial cells (TECs), plays a central role in the establishment and the maintenance of the thymic microenvironment that supports T cell maturation and selection [2, 3]. TECs can be identified by the expression of several proteins [4], including the transcription factor FOXN1, which, in the thymus, is expressed only in TECs [5].

After a phase of rapid growth, the thymus enters a process of involution, which results in the generation of fewer naïve T cells and immunosenescence [6, 7]; thymic involution can also be caused by pathologic conditions and medical treatments (e.g. bone marrow transplant) [8, 9]. The mechanisms controlling thymic development and involution are still poorly understood, limiting the development of therapeutic approaches to improve immune function in a wide variety of patients [6]. For the most part, the pathways that have been implicated in thymic growth are growth-promoting pathways, including signaling downstream of the keratinocyte growth factor KGF and Interleukin 22 [10–12]. Recently, we identified the retinoblastoma tumor suppressor protein (RB) as playing a central role in TECs to regulate thymus size and function, including the production of T cells; these experiments showed that loss of RB family function results in increased TEC proliferation while preserving the differentiation status and the functional properties of TEC populations [13]. These observations raised the possibility that loss of tumor suppressors may provide a novel strategy to promote thymic function.

PTEN (Phosphatase and Tensin Homologue) normally restricts activity of the PI3 Kinase (PI3K)/ Protein Kinase B (PKB, also known as AKT) signaling pathway [14, 15]. In many cases, the PI3K/AKT pathway responds to mitogenic stimuli, activating an intracellular phosphorylation cascade that results in cellular proliferation and increased survival. In its role as a suppressor of these mitogenic signals, PTEN is an important tumor suppressor. Loss of PTEN has been observed in several tumor types, including, but not limited to breast and brain tumors [16, 17]. PTEN also plays a role in the inhibition of cellular migration [18]. Inherited inactivation of PTEN has been implicated in Cowden syndrome, which is characterized by hyper proliferation and benign and malignant tumors [19, 20]. However, loss of PTEN function is not always tumorigenic and it has also been implicated in enhanced tissue repair and regeneration [21–24].

Here we sought to investigate the consequences of PTEN deficiency in TECs upon thymic function. We initially hypothesized that similar to loss of function of the RB pathway, loss of PTEN may stimulate the proliferation of functional TECs and possibly boost thymic function. However, we found that *Pten* deletion in mouse TECs significantly disrupts thymic architecture and function, identifying a key role for PTEN in the thymic epithelium but also suggesting that strategies aiming at reducing PTEN levels and/or activity may not be appropriate to stimulate thymic function.

## Materials and Methods

### Animals

All mice were housed in the Stanford University School of Medicine Research Animal Facilities in accordance with institutional and National Institutes of Health guidelines. All animal care and experiments were approved by the Stanford University Administrative Panel on Laboratory Animal Care. Mice were of a mixed C57BL/6;129Sv/J background. *Foxn1-Cre* mice were a gift from Nancy Manley (University of Georgia) [25]. *Pten*^*Lox/Lox*^ mice (*Pten*^*tm1Hwu*^, [26]) were purchased from the Jackson Laboratories (Bar Harbor, ME).

### Histology and Immunostaining

Hematoxylin and eosin staining (H&E) and trichrome staining were performed on paraffin embedded sections. Thymi were fixed with 4% paraformaldehyde overnight, dehydrated in gradient ethanol solution and embedded in paraffin. 5 μm sections were cut and stained with H&E or with trichrome stain. Microscopy was performed on a DM2000 microscope (Leica) using a DFC500 camera (Leica). Images were captured using Leica LASV3.8 software and processed on ImageJ or Adobe Photoshop software.

Immunofluorescence was performed on 5 μm sections cut from paraffin embedded tissue prepared as described above. Images were obtained using a confocal LSM 510 Meta (Carl Zeiss) with a Plan-Apochromat 20x/0.8 objective and analyzed with AxioVision 4.8 software (Carl Zeiss). Primary antibodies were directed against Foxn1 (goat anti-mouse G-20; Santa Cruz), Keratin 14 (rabbit anti-mouse; Covance), Keratin 8 (rat anti-mouse, Troma I; Developmental Studies Hybridoma Bank), AIRE (rabbit anti-mouse; Santa Cruz) β5T (rabbit anti-mouse; MBL), Involucrin (rabbit anti-mouse; Covance), and Ki67 (mouse anti-human; Pharmingen). Secondary antibodies were purchased from Jackson ImmunoResearch Laboratories, Inc. UEA1 was detected using FITC-UEA1 (Vector Labs).

### Flow Cytometry

T cells were obtained from thymus and spleen by mechanical disruption of the organ to release cells. Red cells were lysed on ice using ammonium lysis buffer. After three cold PBS washes, cells were stained for proteins of interest, washed, and resuspended for analysis in PBS/serum. All flow cytometry was performed either on a BD FACS ARIA II or a LSR Fortessa Analyzer. Data was acquired using FACSDiva (BD) software and analyzed using FlowJo Software (Tree Star). Antibodies for EPCAM, CD25, CD3ε, CD4, CD8, MHCII, CD45, TER119, Mac1, and Gr1 were obtained from eBioscience.

### Statistical Analysis

When appropriate, statistical analysis was performed using two-tailed student’s t-test to compare two populations. A p-value of <0.05 was required for significance (actual p-values are listed in figure legends).

## Results and Discussion

### Loss of PTEN in thymic epithelial cells results in a smaller thymus with disordered architecture

In order to evaluate the effect of loss of PTEN in thymic epithelial cells on thymus size and function, we crossed mice that express the Cre recombinase under the control of the *Foxn1* promoter (*Foxn1-Cre*) with conditional mutant *Pten* mice (*Pten*^*lox/lox*^). This genotype enables the inactivation of the PTEN protein in cells that express *Foxn1*, such as TECs and skin keratinocytes. In the thymus, *Foxn1* is expressed in all TEC populations beginning early during thymic development, with detectable expression as early as embryonic day 11.5 [27, 28]. *Pten* is broadly expressed in mouse tissues (bioGPS.org) and recent microarray data indicate that it is expressed in neonatal TECs [29]. Based on these observations, it is likely that *Pten* is expressed in developing TECs and deleted early during thymic development in *Foxn1-Cre Pten*^*lox/lox*^ mice. In this study, we were interested in studying the consequences of deleting *Pten* during thymic development in the thymus of young mice.

**Fig 1:**
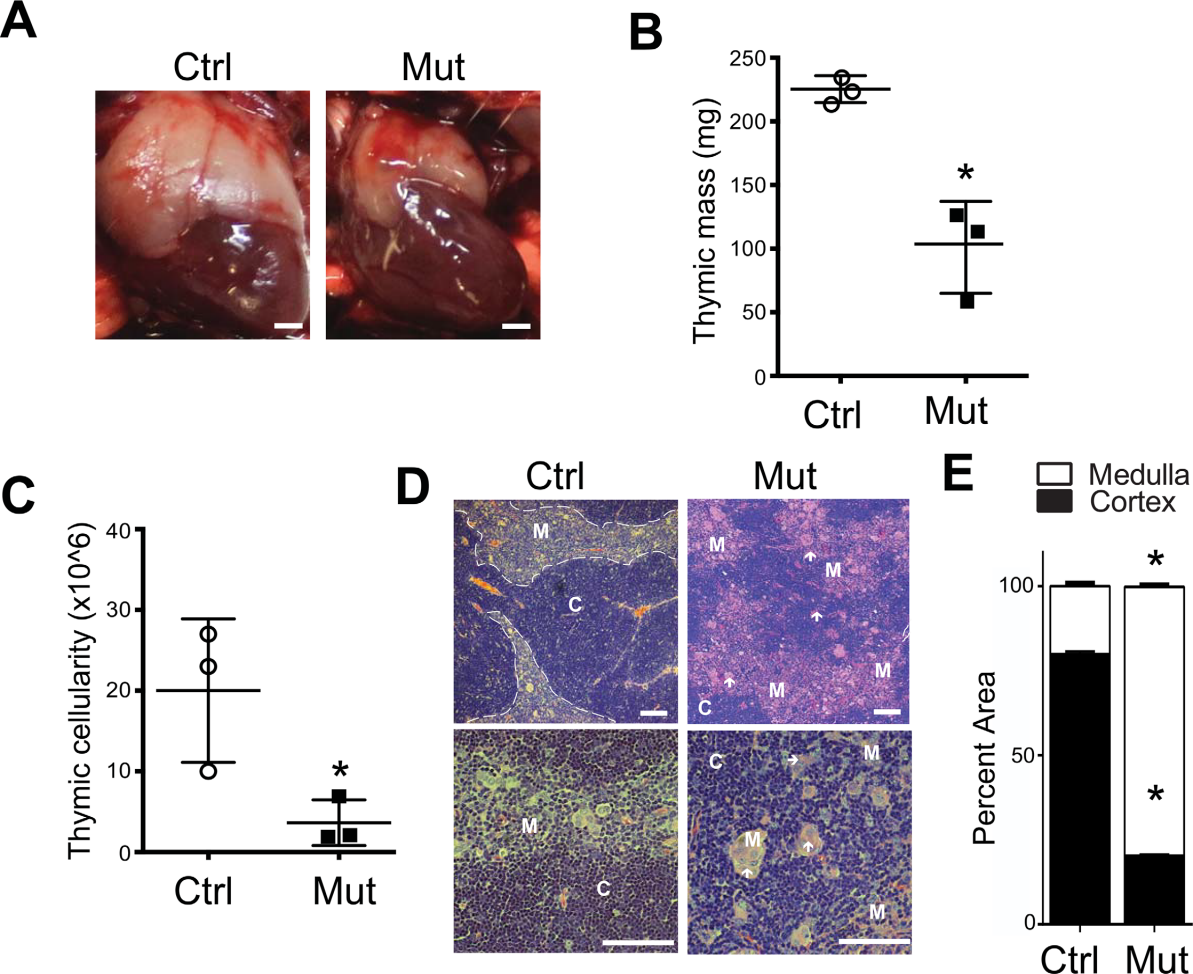
*Foxn1-Cre Pten*^*lox/lox*^ mice have a small thymus with disordered architecture and altered histologic features. (**A**) Representative photographs of thoracotomies performed in 3-week old control (Ctrl, *Pten*^*lox/lox*^) and *Foxn1-Cre Pten*^*lox/lox*^ mutant (Mut) littermate mice. Scale bar, 2500 μm. (**B**) Mass of thymi dissected from control and *Foxn1-Cre Pten*^*lox/lox*^ mutant mice (Control = 212mg ± 11mg; Mutant = 103mg ± 42mg; n = 3 each; p-value = 0.01; 3-week old). (**C**) Quantification of the cellularity in thymi from control and *Foxn1-Cre Pten*^*lox/lox*^ mutant mice (Control = 21.6 x 10^6^ ± 9.6 x 10^6^ cells; Mutant = 4.8 x 10^6^ ± 3.8 x 10^6^ cells; n = 3 each; p value = 0.03; 3-week old). (**D**) Representative images from Hematoxylin and Eosin staining of sections from 3-week old control and mutant thymus. C, cortical; M, medullary. Of note are the large rafts of cells seen in the medulla of mutant mice (white arrows).Scale bar, 100 μm. Hematoxylin and Eosin (H&E) staining from control and mutant thymus. (**F**) Analysis of area represented by cortex and medulla in three non-adjacent, mid-thymus, low-power field from three control and three *Foxn1-Cre Pten*^*lox/lox*^ mutant three week old mice (Cortex: Control = 79.8% ± 0.8%; Mutant = 20.5% ± 0.5%; p <0.0001; Medulla: Control = 20.2% ± 0.8%; Mutant = 79.5% ± 0.5%; p <0.0001).

Upon dissection 3 weeks after birth, we found that *Foxn1-Cre Pten*^*lox/lox*^ mice had a visibly smaller thymus than their control littermates (Fig 1A); no visible size differences were observed in *Foxn1-Cre Pten*^*lox/+*^ mice (not shown) and these heterozygote mutant mice were not studied further. While control thymus weighed approximately 225mg and on average contained around 2 x 10^7^ cells, the thymus from the homozygous mutant mice weighed significantly less than the thymus from control mice, averaging approximately 100mg with almost 4 times fewer cells (approximately 6 x 10^8^; Fig 1B and 1C) than control littermates (Fig 1B and 1C). As expected, we found by histologic examination of thymic sections from three-week-old control mice with hematoxylin and eosin (H&E) staining the thymic cortex densely packed with cells. Similarly, the medulla was also rich in cells but to a lesser density than the cortex; there was a clear, well-defined demarcation between the cortex and the medulla (Fig 1D). The thymi of mutant mice, however, have decreased overall cellularity, an altered cortical:medullary ratio, and increased eosinophilic staining in the medulla (Fig 1D). The demarcation between the cortex and medulla was also disrupted and less distinct. In addition, rather than having the relatively uniform appearance of control thymi, the medulla of *Foxn1-Cre Pten*^*lox/lox*^ mice had several rafts of large, palely staining medullary cells scattered throughout and it was often difficult to find both the cortex and medulla in any given section (Fig 1D).

In order to gain at least a semi-quantitative appreciation of the effects that loss of PTEN from TECs has on the cortical/medullary balance in the thymus, we determined the relative area of the cortex and medulla in three non-adjacent, low-power fields, images taken from mid-thymus of at least three control and three mutant three-week old animals. The cortical:medullary ratio was inverted in mutant mice. In control mice, cortex accounted for approximately 80% of the area, in *Foxn1-Cre Pten*^*lox/lox*^ cortex accounted for only 20% of the area of the thymus. While this approach is semi-quantitative, and the cortical:medullary ratio may be dynamic throughout the life of the mutant animal, this observation underscores the significant disruption of thymic architecture upon *Pten* deletion in TECs. The end result of these effects is not a hyperproliferative thymus, as might be expected following the loss of a key anti-proliferative protein like PTEN, but rather is a smaller, disordered, less cellular thymus.

### Abnormal thymic epithelial architecture in *Pten*-mutant mice

**Fig 2:**
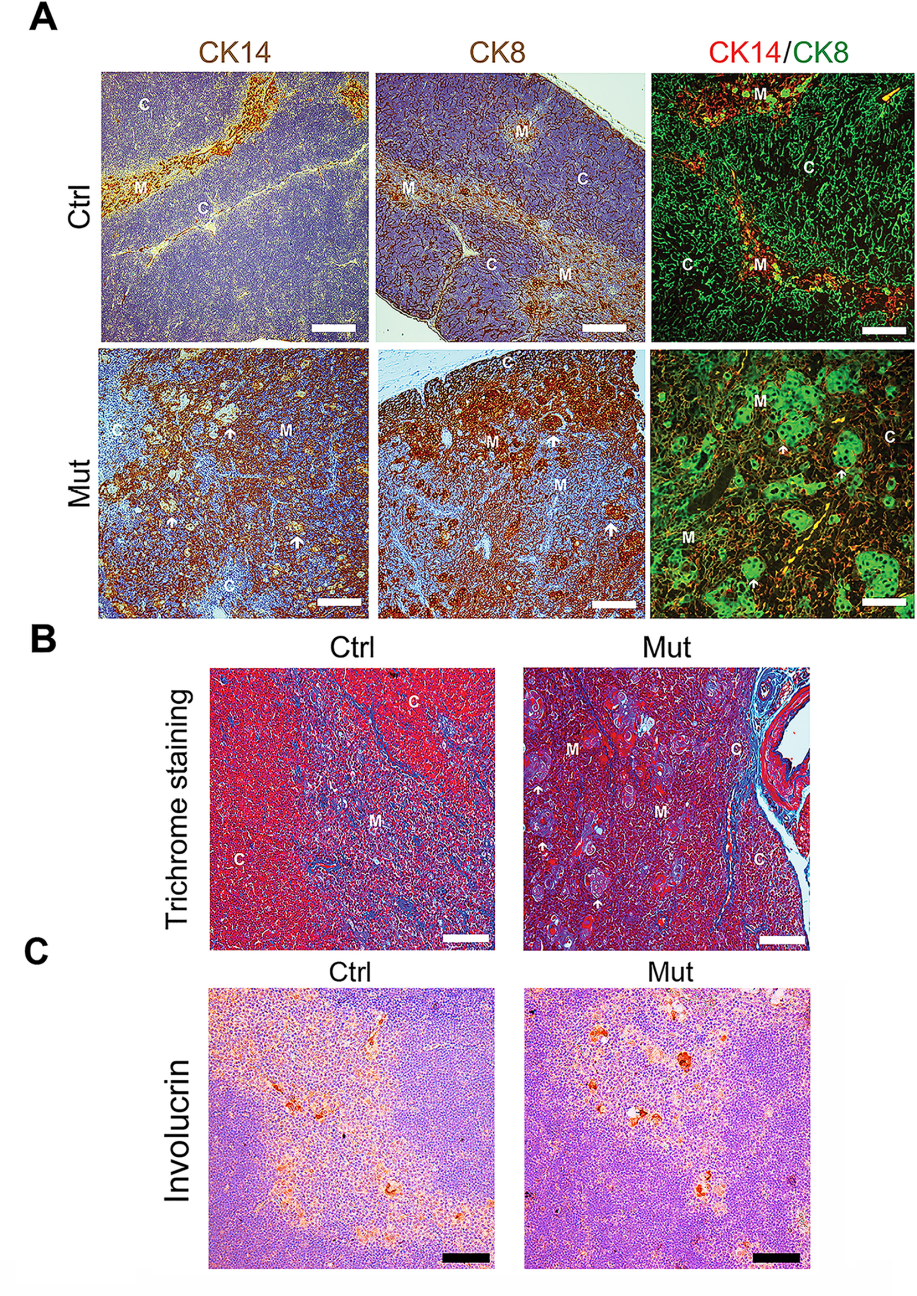
Disruption of epithelial structures in the thymus of *Foxn1-Cre Pten*^*lox/lox*^ mice. (**A**) Left and middle panels: immunostaining of CK14 (medullary TECs) and CK8 (cortical TECs) expression in thymus section from control (*Pten*^*lox/lox*^) and *Foxn1-Cre Pten*^*lox/lox*^ mice. Note that, while there is a clear demarcation between CK14- and CK8-expressing territories in control thymus, mutant thymi have patchy areas of CK expression with less clear demarcation of the cortico-medullary junction. Also of note, the large rafts of cells in the medulla of the thymus stain very strongly for CK8. Right panels: Immunofluorescence analysis of CK expression (CK14, red, and CK8, green) in control and mutant mice. (**B**) Masson’s trichrome staining of 3-week old control and mutant thymus indicates that much of the altered architecture, and especially that of the large cellular rafts, is due to keratinization (red), rather than collagen (blue) or hypercellularization (pink). Scale bar, 100 μm. White arrows indicate large cellular rafts seen in mutant thymus. C = cortical. M = medullary. Pictures shown are representative of at least 3 mice analyzed. Scale bar, 100 μm. (**C**) Staining for Involucrin in sections from 3-week old control and mutant mice demonstrate that both have clusters of positive cells in the medulla of the thymus. In the mutant thymus, these clusters are larger and correspond to the large cell-rafts described above. Scale bar, 100 μm.

Loss of *Pten* may have many effects on the thymus, including, but not limited to disruption of TEC intrinsic signaling and differentiation, alterations in the microenvironment disrupting stromal and T cell function and interaction, and alterations in both TECs and the microenvironment that result in abnormal thymocyte proliferation, development, and selection. We sought to further examine the effects of PTEN loss on cellular differentiation in the developing thymic epithelium. To this end, we took advantage of the fact that cortical TECs (cTECs) and medullary TECs (mTECs) have distinct molecular markers. For example, mTECs predominantly express Cytokeratin 5 and 14 (CK5 and CK14) while cTECs predominantly express Cytokeratin 8 (CK8) [30]. To determine whether there were alterations in Cytokeratin expression patterns in *Pten*-deficient TEC populations, we first performed immunohistochemistry analysis for the respective Cytokeratins. As expected, we found that CK14 was specifically expressed in the medulla in control mice, while CK8 expression was restricted to the thymic cortex. In contrast, we observed intermingling of Cytokeratin 8 and 14 expression in the thymus of *Foxn1-Cre Pten*^*lox/lox*^ mice (Fig 2A). Moreover, even though they are found in the medulla and upon H&E staining appear to be medullary, the rafts of cells in the mutant thymus stained strongly positive for the cTEC marker CK8 and did not express detectable the mTEC marker CK14 (or CK5, not shown). Rather, these rafts of cells were surrounded by regions of dense CK14 staining.

To further investigate whether this breakdown in regional partitioning of Cytokeratin expression was due to disordered architecture or to aberrant expression of these differentiation markers in individual cells, we next performed immunofluorescence analysis of CK8 and CK14 on sections of control and mutant thymus. In control thymi, while there were a few cells in the medulla that expressed CK8, almost every cell appeared to express either CK8 (green) or CK14 (red), as expected (Fig 2A). In *Pten* mutant thymi, these immunofluorescence analyses recapitulated the disordered structure observed by histology and indicated that there are some CK14 cells that also express CK8. While CK14 expressing cells are clearly, and strongly positive in the control mice, double staining analyses were suggestive of some level of CK8 staining in a number of CK14+ TECs. There were very few, if any cells that were as strongly singly positive for CK14 in the thymus of mutant mice. Thus, there may be alterations in the pathways of differentiation into TEC subtypes in Pten mutant mice that would be worthy of pursuit in future investigations.

Masson’s trichrome provides an alternative way to define the medullary and cortical regions of the thymus [31]. With this stain, Keratins are marked in red, collagen stains blue, nuclei stain dark, and the cytoplasm of cells stains light red or pink. Staining further confirmed the disorganization of thymic architecture in *Pten* mutant thymus sections (Fig 2B). The enlarged cells in the mutant thymus showed a combination of enlarged cytoplasm with areas of significant keratin accumulation in many of these cells, indicating that these cells are indeed hyperkeratotic (Fig 2B). Of some peculiar interest, however, not all of these cells stained bright red (Keratin) under trichrome staining, while they all appeared to accumulate antibody against Cytokeratin 8 or 14 (Fig 2A). It is not clear if this discordance reflects alterations in structure or organization of the Keratins in these cell populations.

To further explore what these large cell rafts might represent, we tested the hypothesis that they may be abnormal Hassall’s corpuscles. Hassall’s corpuscles are found in the medulla, are composed of terminally differentiated TECs, and can be identified by involucrin expression [32]. To determine whether these cell rafts are Hassall’s corpuscles, we stained control and mutant thymus from 4-week old mice for Involucrin. Indeed, control thymus had cell clusters of Involucrin-positive cells forming Hassall’s corpuscle-like structures seen in other reports. The large cellular rafts seen in the medulla of mutant mice also stained positive for Involucrin (Fig 2C). While both control and mutant thymus have observable Involucrin-positive cell clusters, they are clearly larger in mutant thymus. Perhaps these rafts are enlarged, potentially abnormal, but terminally differentiated TECs that compose Hassall’s Corpuscle-like structures. The significance of this finding is at this time unclear but is further suggestive of developmental defects in thymic epithelial populations.

**Fig 3:**
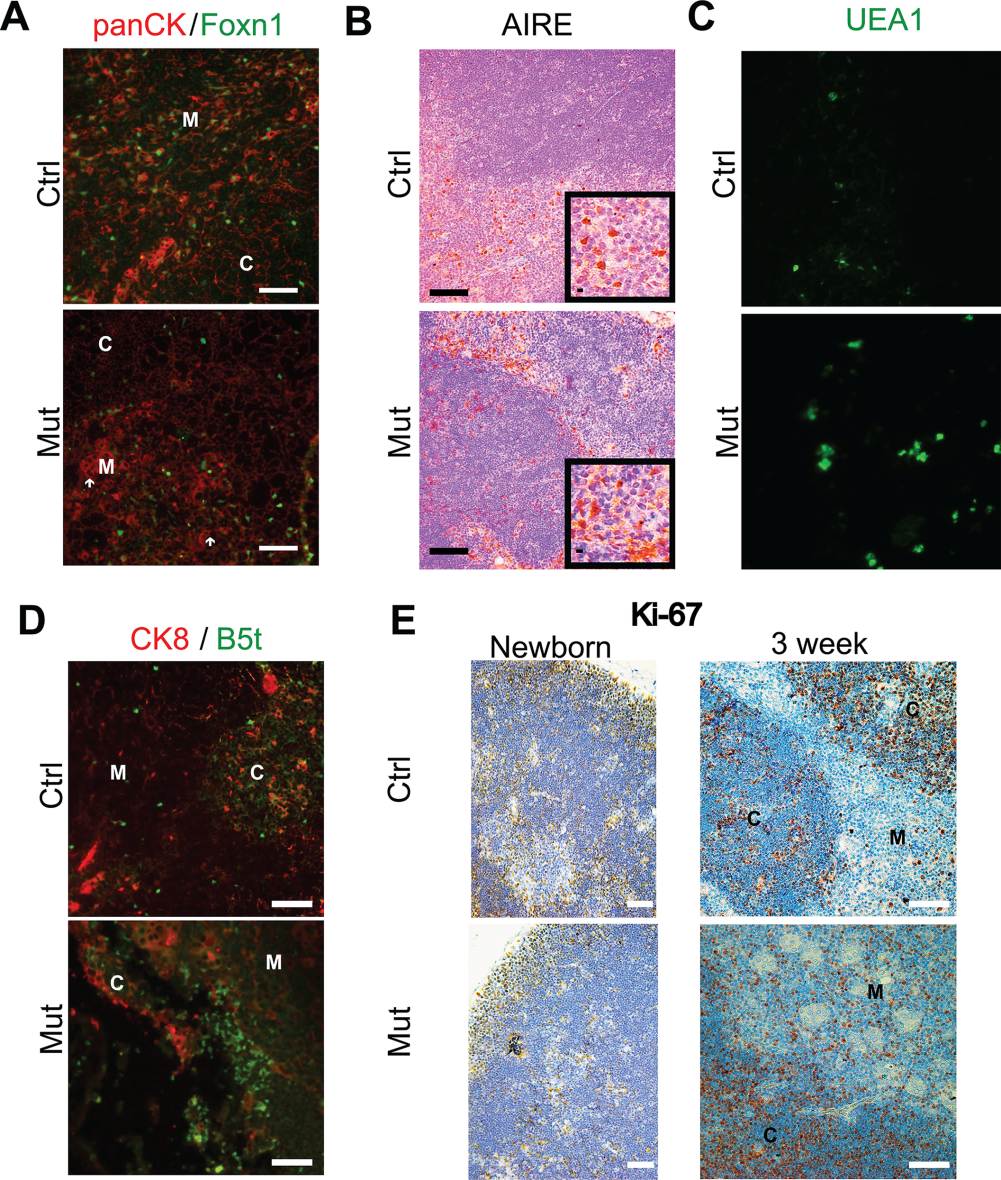
Alterations in the expression of regional differentiation markers and proliferation in the thymus of *Foxn1-Cre Pten*^*lox/lox*^. (**A**) Expression of Foxn1 and cytokeratin using a panCK-directed antibody in the thymus of 3-week old control and *Foxn1-Cre Pten*^*lox/lox*^ mice. White arrows indicate large cellular rafts seen in mutant thymus. Scale bar, 100 μm. (**B**) Expression of the mTEC marker AIRE in the thymus of 3-week old control and *Foxn1-Cre Pten*^*lox/lox*^ mice. Inset images are high-power magnification of select regions of the larger image. Scale bars, 100 μm (**C**) Expression of the mTEC marker UEA1 in the thymus of 3-week old control and *Foxn1-Cre Pten*^*lox/lox*^ mice. Scale bars, 100 μm (**D**) Left: cortical expression of the β5t proteasomal subunit (immunofluorescence, green) in the thymus of 3-week old control and *Foxn1-Cre Pten*^*lox/lox*^ mice. CK8 staining (red) marks cortical TECs. Scale bars, 100 μm. (**E**) An evaluation of cellular proliferation as indicated by Ki-67 staining in the thymus of newborn (left panels) and 3-week old control and *Pten* mutant mice. Scale bars, 100 μm.

As Foxn1 is a critical component and early driver of TEC proliferation, differentiation, and survival programs [33, 34], we wondered whether loss of PTEN may affect Foxn1 expression and examined Foxn1 expression in the thymus of wild-type and mutant mice. Staining for Foxn1 in control and *Foxn1-Cre Pten*^*lox/lox*^ thymus demonstrated clear expression in both control and mutant thymi indicating that PTEN is not necessary for continued Foxn11 expression (Fig 3A). It must be noted that the Cre recombinase is driven by a *Foxn1* promoter, so *Pten* is not deleted until after *Foxn1* expression has begun in these mutant mice. It is possible that earlier deletion of the *Pten* locus could prevent or otherwise alter *Foxn1* expression.

Because of the significant changes in the architecture of the medulla in the mutant mice, we investigated whether there were alterations in the expression pattern of two medullary expressed markers–AIRE [35] and UEA1 [36]. AIRE expression is restricted to a subset of mTECs in the thymus control mice (Fig 3B). Moreover, staining is predominantly nuclear (inset, upper panel, Fig 3B). Meanwhile in *Pten* mutant mice, there appeared to be increased numbers of AIRE positive cells in the medulla, when qualitatively compared to controls. In addition there were many AIRE-positive cells in the cortex, supporting that there are either cell-intrinsic cues that disrupt the appropriate geographic expression of AIRE, or perhaps that there are disrupted cues in the microenvironment that lead to ectopic expression of AIRE (Fig 3B, lower panel). Moreover, AIRE staining is more diffuse, speckled, and more prominent in the cytoplasm within cells of the mutant medulla, suggesting that PI3K/AKT activity, or perhaps PTEN itself play a role in the subcellular localization, and potentially, the regulation of AIRE (inset, lower panel, Fig 3B).

UEA1 expression patterns were more visibly affected in the mutant mice, with enhanced expression in the enlarged, hyperkeratotic, Involucrin-positive mTEC populations. There also appeared to be, qualitatively, an increase in UEA1 expression in the medulla of *Pten* mutant mice (Fig 3C). UEA1 expression, however, remained confined to the medulla in both control and mutant mice. These observations suggested that loss of PTEN may affect levels, if not localization of UEA1, but it is not clear from these initial investigations what the significance of this increase in UEA1 expression implies for thymus architecture and function.

To begin to examine whether there are similar alterations in protein expression patterns in the cortex of *Foxn1-Cre Pten*^*lox/lox*^ thymus, we determined the expression pattern of a marker of a subset of cTECs. Select cTECs expresses the β5t subunit of the proteasome [37, 38]. We investigated the expression of this marker in the thymus of control and mutant mice. In the control thymus, we found well-dispersed occasional β5t expression throughout the cortex and only in the cortex (Fig 3D), as reported. Meanwhile, in mutant mice, β5t expression remained restricted to the cortex, but was densely clustered in the reduced rim of cortex, perhaps due to increased numbers of cells expressing β5t or to the loss of cells that do not express β5t (Fig 3D).

It is possible that many of the alterations seen in thymic architecture and expression patterns are not primary results of the loss of PTEN, but rather secondary effects due to alterations in stromal and T cell proliferation and regional or total cellularity. For instance, abnormally large or apparently ectopic groups of cells expressing a particular marker may not be grouping together due to altered migration clues that draw them together, but rather due to loss of cells that would otherwise be separating the abnormal groups of cells in a normal thymus. To begin to test this hypothesis, we determined whether there were observable differences in proliferation–both global and regional–in the *Foxn1-Cre Pten*^*lox/lox*^ thymus compared to the control thymus. To do so, we stained thymus from newborn and three-week old control and *Foxn1-Cre Pten*^*lox/lox*^ mice for the proliferation marker, Ki-67 and found that while there were no striking differences in Ki-67 staining in newborn mice, there was an easily apparent increase in medullary Ki-67 signal in mutant thymi (Fig 3E). This increase in proliferative cells in the medulla in *Pten* mutant mice certainly can explain the increase in medullary area in the mutant thymus, but it does not seem to account for the other alterations in architecture. By itself it does not explain the overall decrease in size and cellularity seen in mutant mice. Rather, it can only suggest that the alterations in TEC and regional architecture might not support normal thymocyte numbers and even though there is an increased proliferative rate, the result is a decrease in overall size and cellularity. We have not examined cell death in epithelial and non-epithelial thymic populations, but increased cell death rates may account for the decrease in overall size.

The presence of Foxn1 and other key markers of medullary and cortical TECs indicate that loss of Pten does not completely disrupt differentiation programs in the developing thymic epithelium. However, some of the patterns of expression noted correlate with the disorganized thymic architecture and indicate that there is disruption of the normal thymus in the absence of Pten from TECs. The exact nature of these defects will require additional studies, including evaluation of additional thymus differentiation makers (e.g. CD80, MHCII) in these mice, description of the thymus in these mice during embryonic development, and deletion *Pten* at different times during thymic development and in different subsets of TECs. Moreover, studies of the intracellular signaling cascades and intercellular communication might be of high interest for future investigations.

### Decreased T-cell production and altered CD4/CD8 ratio in *Foxn1-Cre Pten*^*lox/lox*^ mice

**Fig 4:**
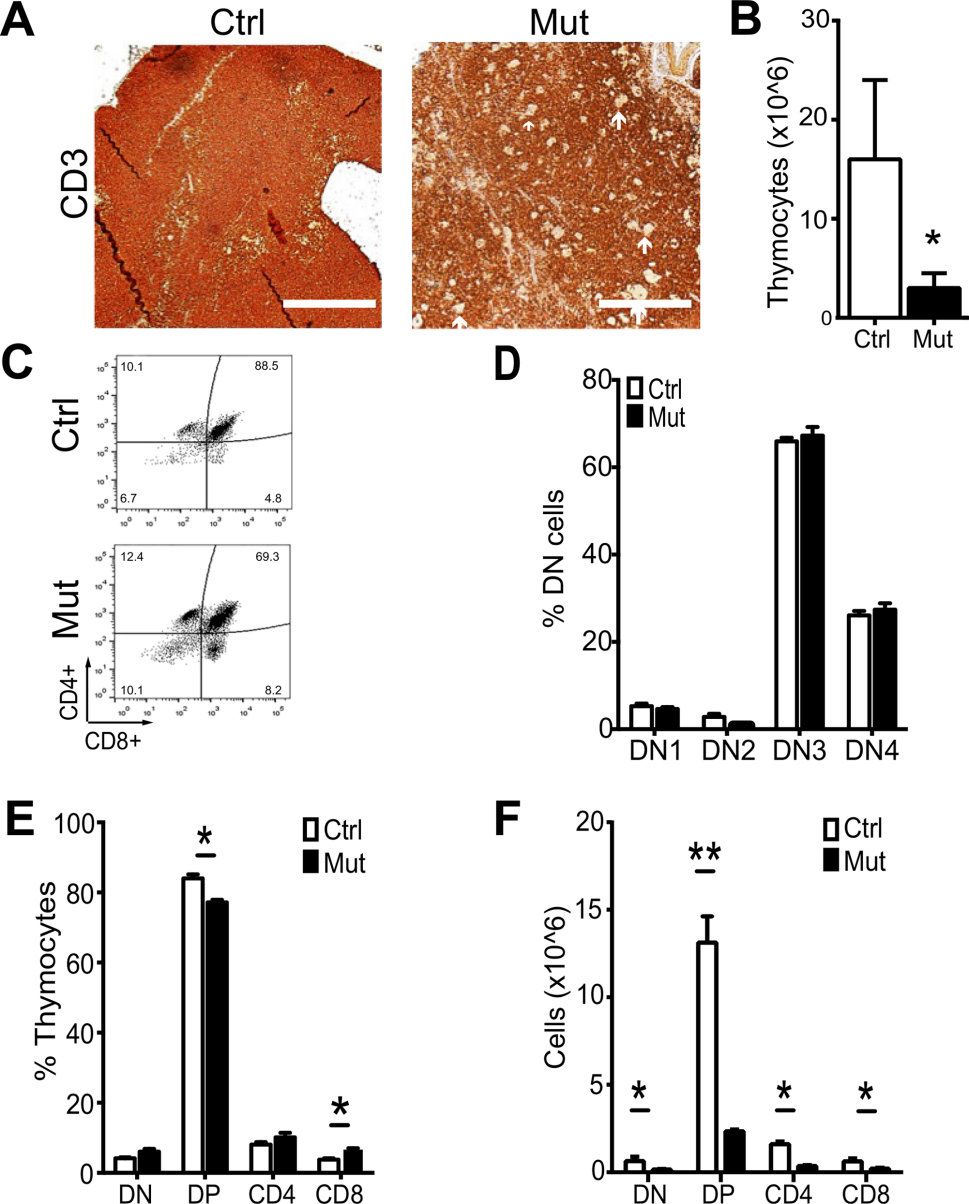
Defects in Thymocyte populations in 3-week old *Foxn1-Cre Pten*^*lox/lox*^ mice. (**A**) Immunohistochemical staining of control and mutant thymus for the pan-T cell marker CD3. Controls (staining of non-hematopoietic organs and secondary antibody only on thymus sections) confirmed the specificity of the staining (data not shown). (**B**) Calculated number of thymocytes (total cellularity times percent CD3+ cells per thymus) in control and *Foxn1-Cre Pten*^*lox/lox*^ thymus (n = 3 each; p = 0.045, asterisk indicates significance). (**C**) Representative flow cytometry of DN (CD4^-^/CD8^-^), DP (CD4^+^/CD8^+^), CD4^+^ and CD8^+^ thymocytes in control and *Foxn1-Cre Pten*^*lox/lox*^ mice. (**D**) Quantification of DN T cell subsets in control and *Foxn1-Cre Pten*^*lox/lox*^ mice (n = 4; p values = DN1 = 0.20, DN2 = 0.12, DN3 = 0.39, DN4 = 0.34). (**E**) Quantification of T cell subsets in control and *Foxn1-Cre Pten*^*lox/lox*^ mice (n = 4; p values = DN = 0.25, DP = 0.01, CD4 = 0.30, CD8 = 0.03; asterisks indicate significance). (**F**) Calculated numbers of total T cell numbers in control and mutant mice (n = 4; p values = DN = 0.01, DP < 0.01, CD4 = 0.01, CD8 = 0.03; asterisks indicate significance). Additional quantification data related to this figure can be found in Table 1.

The primary function of the thymus is to produce functional T cells. Thus we examined whether loss of PTEN in TECs may affect T cell production, resulting in the smaller, less cellular thymus. First, we performed immunohistochemistry against the pan-T cell marker CD3. T cells comprise the vast majority of cells in the thymus and, in controls; we observed very dense CD3 staining throughout the thymus of these animals, with relatively increased density of T cells in the cortex compared to the medulla, as expected. Meanwhile, CD3 staining was less dense in the thymus of mutant mice, in particular, within the medulla, suggestive of overall decreased numbers of T cells (Fig 4A) and absence of T cells from the clusters of terminally differentiated TECs (Hassall’s Corpuscle-like structure). The decrease in T cell numbers was confirmed by flow cytometry (Fig 4B). These observations correspond with and explain, at least in part, our initial observations that the *Foxn1-Cre Pten*^*lox/lox*^ thymus is smaller and less cellular than is thymus for control littermates (Fig 1A–1C).

**Table 1:** Quantification of T cell subsets in the thymus of control and *Foxn1-Cre Pten*^*lox/lox*^ mutant mice (related to Fig 4).

Thymocyte Subset	Analysis	Control	Mutant	p-value
Total				
	Cell Count	16.8 x 10^6^ ± 11.3 X 10^6^	4.1 x 10^6^ ± x 1.9 x 10^6^	0.045
DN1				
	Percentage	6.1% ± 0.4%	5.8% ± 0.2%	0.20
DN2				
	Percentage	4.2% ± 0.6%	0.9% ± 0.4%	0.12
DN3				
	Percentage	66.7% ± 1.6%	68.1% ± 3.6%	0.39
DN4				
	Percentage	25.2% ± 1.8%	27.2% ± 2.6%	0.34
DN				
	Percentage	3.9% ± 0.6%	5.1% ± 0.2%	0.25
	Cell Count	0.7 x 10^6^ ± 0.3 x 10^6^	0.01 x 10^6^ ± x 0.01 10^6^	0.01
DP				
	Percentage	83.4% ± 2.2%	77.3% ± 1.2%	0.01
	Cell Count	13.6 x 10^6^ ± 1.9 x 10^6^	3.2 x 10^6^ ± 0.9 x 10^6^	<0.01
CD4				
	Percentage	9.6% ± 1.7%	11.1% ± 3.3%	0.30
	Cell Count	1.7 x 10^6^ ± 0.2 x 10^6^	.05 x 10^6^ ± 0.1 x 10^6^	0.01
CD8				
	Percentage	3.3% ± 0.5%	6.6% ± 1.2%	0.03
	Cell Count	0.6 x 10^6^ ± x 0.2 x 10^6^	0.3 x 10^6^ ± 0.1 x 10^6^	0.03

Next, we sought to characterize whether there was a generalized loss of T cells in the thymus or rather, if there was a loss of particular thymocyte subset. Thymocytes develop through a well-described set of developmental stages that can be described both by their cell surface marker expression and roughly approximated by their geographical location in the thymus. Briefly, thymic precursors enter the thymus at the cortico-medullary junction. They then migrate through the cortex through a series of four double negative (DN: CD4- CD8-; DN sub-stages are DN1: CD44+ CD25-; DN2: CD44+ CD25+; DN3: CD44+ C25-; DN4: CD44- CD25-) stages. Thymocytes mature to the double positive stage (DP: CD4+ CD8+). In addition, during this migration through the cortex, the T cells undergo positive and negative selection after which they return to the medulla as single positive T cells (CD4+: CD3+ CD4+ CD8- or CD8+: CD3+ CD4- CD8+) where they further mature prior to exiting the thymus and returning to the circulation [27].

We used this understanding of intrathymic T cell maturation to determine whether there were alterations in the representation of any of the intrathymic T cell populations and quantified the percentage of DN, DP, CD4 and CD8 cells in the thymus of control and mutant mice (Fig 4C). We found no significant differences in the percentages of these DN subsets present between control and mutant mice (Fig 4D). Likewise, there were no significant differences in percentage of total DN cells between control and mutant mice. While control and mutant mice had similar percentages of CD4+ T cells in the thymus, there was a significant decrease in percentage of DP cells present and a significant increase in the percentage of CD8 cells in the thymus of mutant mice compared to controls (Fig 4E). There was, however, a decrease in total cell numbers across all four major subsets of T cells in *Pten* mutant thymus compared to control thymus, with the most striking difference found in the DP cell population (Fig 4F). Thus the *Foxn1-Cre Pten*^*lox/lox*^ thymus is not able to support as many thymocytes as is the control thymus and there is either a shortening of transit time through DP or alterations in the selection and support of DP cells. Whether the increase in CD8 cells is due to a preferential trend towards CD8 cells within the DP population or selection of CD8 cells is more permissive in *Foxn1-Cre Pten*^*lox/lox*^ thymus cannot be answered by the experiments we have performed here but is certainly worthy of future study.

**Fig 5:**
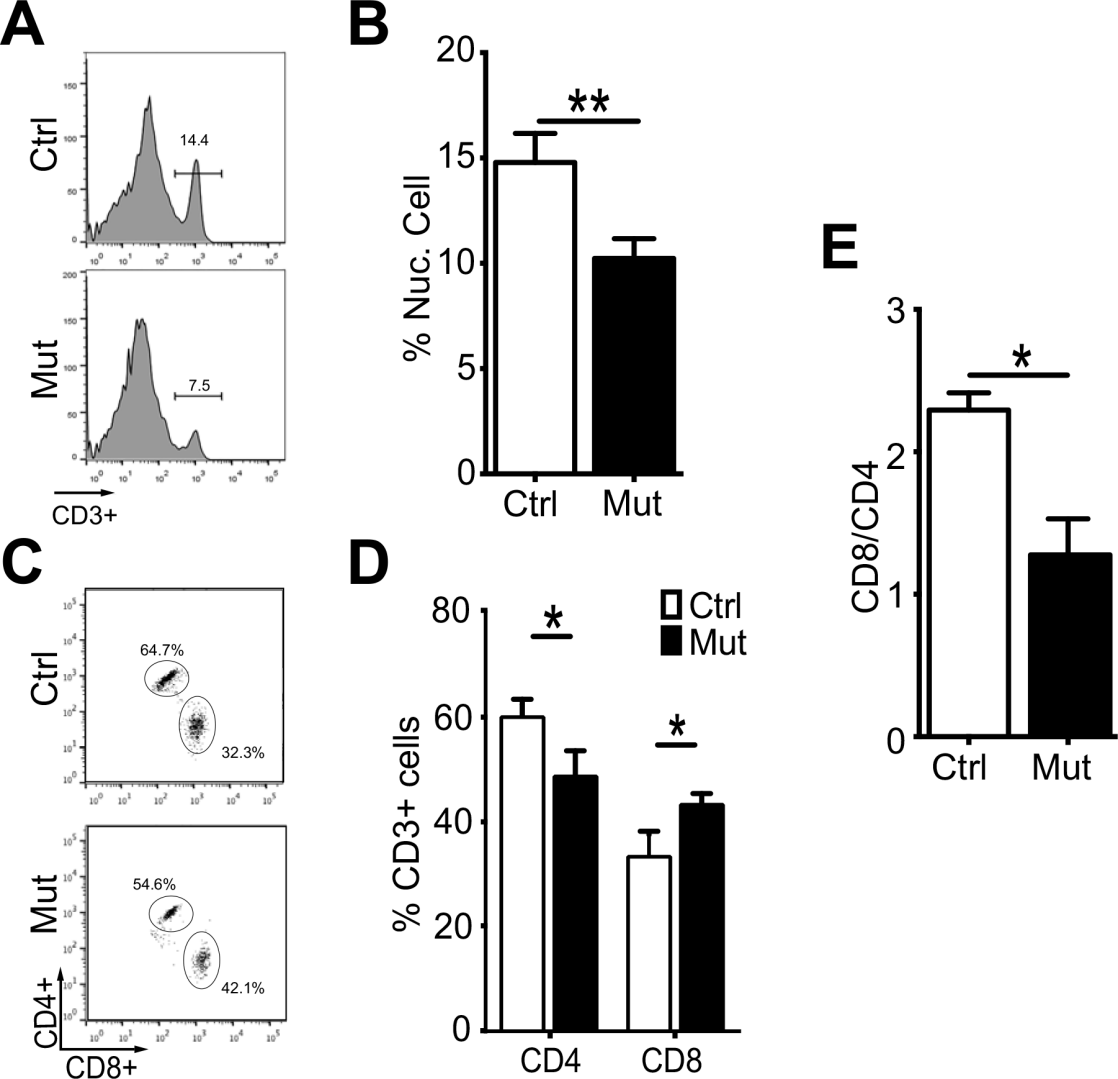
Changes in peripheral T cell populations in the spleen of *Foxn1-Cre Pten*^*lox/lox*^ mutant mice. (**A**) Representative flow cytometry of CD3+ cells (bracketed population) as compared to all nucleated cells found in the spleen of control and *Foxn1-Cre Pten*^*lox/lox*^ mice. (**B**) Quantification of (A) (n = 4; p = 0.00). (**C**) Representative flow cytometry of CD4+ and CD8+ CD3+ cells in the spleen of control and *Foxn1-Cre Pten*^*lox/lox*^ mice. (**D**) Quantification of (C) (n = 4; p = 0.03; p = 0.02). (**E**) CD8/CD4 ratio in the spleen of control and *Foxn1-Cre Pten*^*lox/lox*^ mice (n = 4; p = 0.01). Additional quantification data related to this figure can be found in Table 2.

Following development in the thymus, T cells emerge to the periphery. Thus, we next sought to determine whether the changes in the number and type of T cell in the thymus corresponded with similar changes in the periphery. We focused on T cells in the major peripheral lymphoid organ, the spleen. First we determined what percentage of nucleated cells in the spleen were T cells. Approximately 15% of the nucleated cells in the spleen of control animals were T cells. In contrast, only approximately 10% of the nucleated cells in the spleen of *Foxn1-Cre Pten*^*lox/lox*^ mice were T cells (Fig 5A and 5B). Thus, the loss of thymocytes in *Foxn1-Cre Pten*^*lox/lox*^ thymus translates into the loss of splenic, and perhaps total peripheral T cells. Similar to the thymus, there were more CD8 cells in the spleen of mutant mice, which was accompanied by a decrease in the representation of CD4 cells in these spleens (Fig 5C and 5D). This translated into a significant decrease in the CD4/CD8 ratio from slightly more than 2:1 in control spleen to about 1:1 in the spleen of *Foxn1-Cre Pten*^*lox/lox*^ mutant mice (Fig 5E). The alteration in peripheral CD4/CD8 ration may be due to one or more mechanisms including decreased survival of CD4 cells, increased CD8 cells, or differences in CD4 and CD8 T cell egress from the *Foxn1-Cre Pten*^*lox/lox*^ thymus. It would not be surprising to find, however, that there are significant differences in immune function in *Foxn1-Cre Pten*^*lox/lox*^ thymus compared to controls.

**Table 2:** Quantification of T cell subset in the spleen of control and *Foxn1-Cre Pten*^*lox/lox*^ mutant mice (related to Fig 5).

Thymocyte Subset	Analysis	Control	Mutant	p-value
CD3				
	% of nucleated cells	14.9% ± 2.6%	10.2% ± 2.1%	0.00
CD4				
	Percentage	60.0% ± 2.4%	48.7% ± 3.0%	0.03
CD8				
	Percentage	33.4% ± 2.8%	43.3% ± 1.2%	0.02
CD4/CD8				
	Ratio	1.8	1.1	0.01

## Conclusions

We have performed an initial characterization of the consequences of deleting *Pten* in the epithelial compartment of the developing thymus in mice using a TEC-specific *Foxn1* promoter and a *loxP-*flanked *Pten* allele. *Pten* is typically an anti-proliferative gene. Nevertheless loss of *Pten* in TECs results in a smaller, disordered thymus in mice. Expression of several markers of differentiated TECs is altered in *Pten* mutant mice. These findings provide the basis for many additional studies. For example, mTEC development may be divided into stages identifiable by CD80 and MHCII staining [39] and investigation of these markers on mTECs in *Pten* mutant mice may reveal blocked or abnormal mTEC differentiation. This may enable careful further study of the stage of development at which mTECs are halted as well as provide a platform to study the intra- and extracellular programs that drive mTEC development.

On the other hand, expression of the *Foxn1*, does not appear to be significantly altered following loss of *Pten* from TECs, suggesting that PTEN plays a more critical role only later stages of the differentiation program of TECs. It is not clear, however, what the effect of earlier or later deletion of *Pten* might be upon the thymus or what the mechanisms are that are dependent upon *Pten*. That such a dramatic effect results from *Pten* suggests that PI3K, AKT, and other proliferative signals are likely to play a critical, as of yet undescribed role in TEC, thymus, thymocyte, and immune system development and function. Certainly, this could provide many intriguing areas of investigation into the role of PI3K and other kinase cascades in TEC development.

In conjunction with the size and differentiation abnormalities in the thymus of *Foxn1-Cre Pten*^*lox/lox*^ mice, there are alterations in the development of T cells in the thymus, with a shift towards increases in CD8+ cells. This change in thymic T cell development is accompanied by a decrease in the number of T cells in the periphery, but still with an increase in the proportional representation of CD8+ cells compared to control mice. This suggests, at least, that there is a role for kinase signaling in establishing and maintaining T cell selection. While we have studied what the freeing of the PI3K and Akt pathways might have on T cells, we have not yet studied what the effect of increased inhibition of these pathways might do to peripheral T cells numbers and thymocyte development. There are several compounds in the clinic or in clinical trials that modulate the PI3K and Akt pathways. Especially in the advancing age of transplantation, immune-oncology and cell-based therapies, it may become important to determine the effects of kinase cascade modulation has not only on circulating T cells but also on the thymic microenvironment to enable exciting new therapies to achieve the full potential and to minimize their unexpected effects.
